# Genetic Susceptibility to Periodontal Disease in Down Syndrome: A Case-Control Study

**DOI:** 10.3390/ijms22126274

**Published:** 2021-06-10

**Authors:** María Fernández, Alicia de Coo, Inés Quintela, Eliane García, Márcio Diniz-Freitas, Jacobo Limeres, Pedro Diz, Juan Blanco, Ángel Carracedo, Raquel Cruz

**Affiliations:** 1 Grupo de Investigación en Odontología Médico-Quirúrgica (OMEQUI), Instituto de Investigación Sanitaria de Santiago de Compostela (IDIS), Universidade de Santiago de Compostela, 15782 Santiago de Compostela, Spain; maria.fernandez.casado@rai.usc.es (M.F.); eliane.garma@gmail.es (E.G.); jacobo.limeres@usc.es (J.L.); pedro.diz@usc.es (P.D.); juan.blanco@usc.es (J.B.); 2 Grupo de Medicina Xenómica, Centro Singular de Investigación en Medicina Molecular y Enfermedades Crónicas (CIMUS), Universidade de Santiago de Compostela, 15782 Santiago de Compostela, Spain; alicia14coo@hotmail.com (A.d.C.); ines.quintela@usc.es (I.Q.); angel.carracedo@usc.es (Á.C.); raquel.cruz@usc.es (R.C.); 3 Centro Nacional de Genotipado, Plataforma de Recursos Biomoleculares, Instituto de Salud Carlos III (CeGen-PRB3-ISCIII), Universidade de Santiago de Compostela, 15706 Santiago de Compostela, Spain; 4 Centro Singular de Investigación en Medicina Molecular y Enfermedades Crónicas (CIMUS), CIBERER-Instituto de Salud Carlos III, Universidade de Santiago de Compostela, 15706 Santiago de Compostela, Spain; 5 Fundación Pública Galega de Medicina Xenómica—SERGAS, 15706 Santiago de Compostela, Spain

**Keywords:** Down syndrome, periodontitis, genome-wide association study

## Abstract

Severe periodontitis is prevalent in Down syndrome (DS). This study aimed to identify genetic variations associated with periodontitis in individuals with DS. The study group was distributed into DS patients with periodontitis (*n* = 50) and DS patients with healthy periodontium (*n* = 36). All samples were genotyped with the “Axiom Spanish Biobank” array, which contains 757,836 markers. An association analysis at the individual marker level using logistic regression, as well as at the gene level applying the sequence kernel association test (SKAT) was performed. The most significant genes were included in a pathway analysis using the free DAVID software. C12orf74 (rs4315121, *p* = 9.85 × 10^−5^, OR = 8.84), LOC101930064 (rs4814890, *p* = 9.61 × 10^−5^, OR = 0.13), KBTBD12 (rs1549874, *p* = 8.27 × 10^−5^, OR = 0.08), PIWIL1 (rs11060842, *p* = 7.82 × 10^−5^, OR = 9.05) and C16orf82 (rs62030877, *p* = 8.92 × 10^−5^, OR = 0.14) showed a higher probability in the individual analysis. The analysis at the gene level highlighted PIWIL, MIR9-2, LHCGR, TPR and BCR. At the signaling pathway level, PI3K-Akt, long-term depression and FoxO achieved nominal significance (*p* = 1.3 × 10^−2^, *p* = 5.1 × 10^−3^, *p* = 1.2 × 10^−2^, respectively). In summary, various metabolic pathways are involved in the pathogenesis of periodontitis in DS, including PI3K-Akt, which regulates cell proliferation and inflammatory response.

## 1. Introduction

Down syndrome (DS) is the most common chromosomal condition, occurring in approximately one of every 800 live births [1]. DS is associated with intellectual disability and systemic disorders that include heart problems, hematological disorders and endocrinopathies [2]. DS is also characterized by various orofacial manifestations, such as macroglossia, dental malocclusions, delayed tooth eruption and periodontal disease [3].

Individuals with DS have a higher prevalence of periodontitis than that observed in the general population and in other groups with intellectual disability [4], given that a number of studies have reported the condition in more than 90% of patients with DS younger than 30 years [5]. Periodontitis in these patients debuts at an early age and is generalized, progresses rapidly and is severe [6]. The local causes that have been linked to this process include poor oral hygiene, macroglossia, tooth morphology, gingival tissue abnormalities and the characteristics of the saliva [7].

Another important factor that has been related to the etiopathogenesis of periodontal disease in patients with DS is the presence of a subgingival flora different from that exhibited by the general population, such as an increased presence of *Streptococcus gordonii*, *S. mitis* and *S. oralis*, pioneer organisms that start an early microbial colonization, and *Treponema socranskii*, a bacterial species associated with severe destruction of periodontal tissue [8]. It has recently been suggested that *Porphyromonas* spp. and *Tannerella* spp. are particularly abundant in the subgingival microbiome of individuals with DS and periodontitis, as well as new pathogens that have been scarcely studied to date, such as *Filifactor*, *Fretibacterium* and *Desulfobulbus* genera [9].

Moderate B-cell and T-cell lymphopenia, and the reduced response of specific antibodies and neutrophil chemotaxis dysfunction make individuals with DS particularly susceptible to infections attributable to immune system dysfunction, such as periodontal disease [10]. Some studies have suggested that high levels of cytokine involved in systemic inflammation are already detected in children with DS, with significant effects on the immune response regulation, such as tumor necrosis factor alpha, IL-1β and interferon gamma [11,12]. The concentration in the gingival crevicular fluid of some of these cytokines particularly involved in the development of periodontitis is also higher among individuals with DS than in the general population [13].

Other biomarkers have been described that might play a role in periodontal inflammation, such as soluble urokinase plasminogen activator receptor (suPAR), galectin, NOD-like receptor family pyrin domain-containing protein-3 (NLRP3) inflammasome complex, matrix metalloproteinase-8 (MMP-8) and MMP-9 [14,15,16]. These biological markers might help accelerate the development of other systemic inflammatory conditions, such as coronary heart disease [17,18] and diabetes [19], through periodontitis. However, the levels of these biomarkers in the saliva and serum of patients with DS and periodontal disease is still unknown.

Certain genetic factors also play a relevant role in the onset and development of periodontitis, especially in the rapidly progressive presentations, although the genetic bases of this common complex disease have still not been completely determined [20]. However, it has been suggested that the development of periodontitis in individuals with DS responds to a combination of immune system abnormalities that predispose the individual to infections [10] and to an increased migration of T cells towards the periodontium in response to an increase in matrix metalloproteinase levels [21]. Accordingly, the established null hypothesis is that, among individuals with DS, there are no genetic risk factors that favor the onset of periodontitis.

The aim of this study was to detect the genetic variations associated with the presence of periodontitis in individuals with DS and to identify the susceptibility genes and biomarkers that can help predict its risk of onset. Moreover, it could also provide new information on the molecular and genetic mechanisms that affect the start and progression of aggressive periodontitis (stages III/IV, grade C periodontitis) [22].

## 2. Results

Table 1 details the results of the SNPs that showed a greater association with periodontitis (*p* < 5 ×10^−4^) in the logistic regression analysis performed for each SNP (under the dominant model) adjusting for covariates. Of these “top SNPs” and based on the odds ratio values, two would be considered risk SNPs (rs4315121 and rs11060842), and the other three would be considered protective (rs4814890, rs14497874 and rs6203877). However, none of these SNPs reached a significant association value at the genome-wide association study level (*p* < 5 × 10^−8^), and none presented clear evidence of association (*p* < 5 × 10^−6^).

Table 2 shows the results of the genes with greater significance (*p* < 5 × 10^−4^). After conducting the multitest correction, none of these focus genes reached the significance threshold of 2.7 × 10^−6^ for 18,520 analyzed genes.

**Figure 1:**
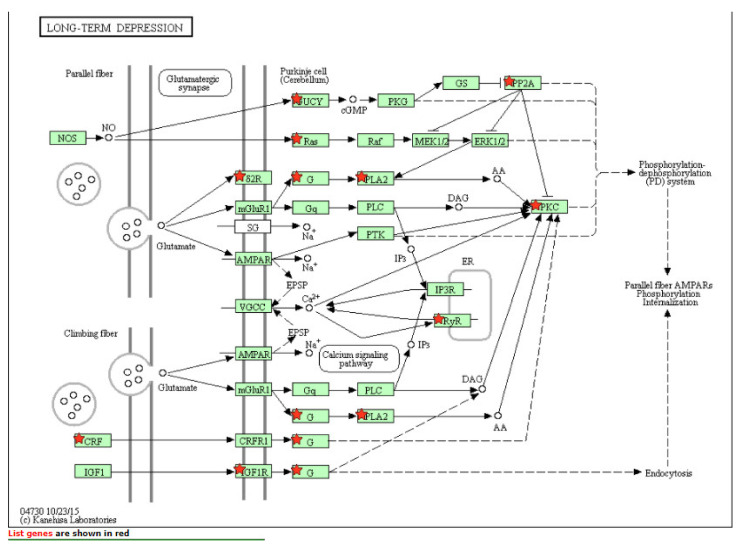
Long-term depression pathway.

**Figure 2:**
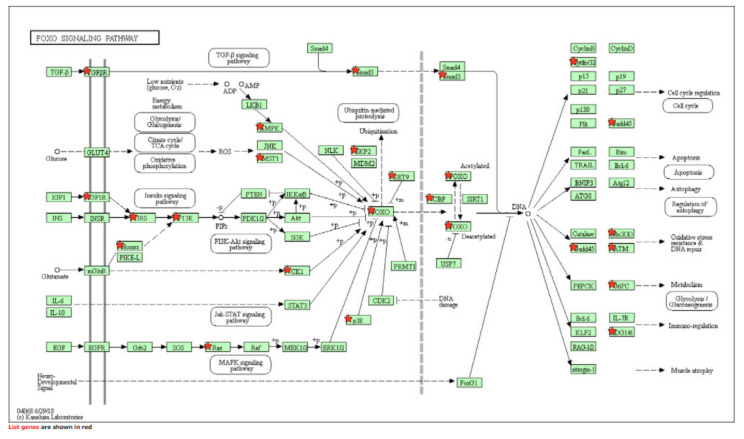
FoxO signaling pathway.

**Figure 3:**
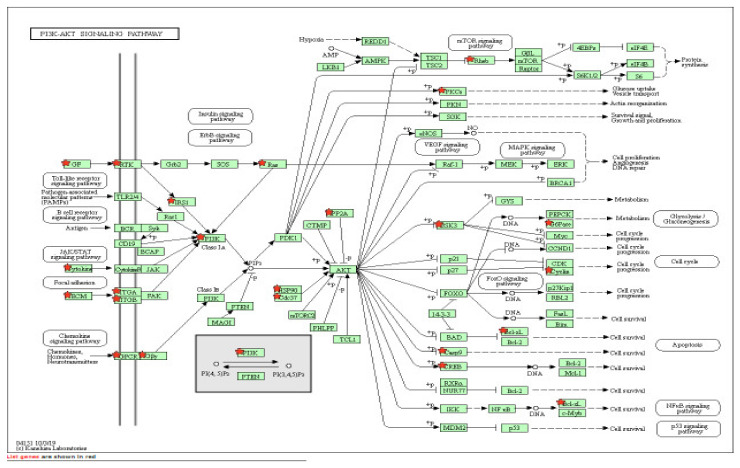
PI3K-Akt signaling pathway.

Table 3 details the list of resulting pathways after the analysis (with DAVID software) of all the genes that showed a *p* < 0.05 in the SKAT analysis at the gene level (*N* = 2464 genes). We obtained 22 pathways (*p* < 0.05) where there was an overexpression of the genes included in the analysis. Those of greatest interest were long-term depression (15 overexpressed genes of those included in the analysis), FoxO signaling pathway (25 overexpressed genes of those included in the analysis) and especially the phosphatidylinositol 3-kinase (PI3K-Akt) signaling pathway (53 overexpressed genes of those included in the analysis) (Figure 1, Figure 2 and Figure 3).

## 3. Discussion

In this study, none of the analyzed SNPs reached a statistically significant association value at the genome-wide association level, probably due to the lack of potency resulting from the small sample size. It should be noted that although we analyzed samples from only 86 participants, the initial study group consisted of 183 individuals with DS, which confirms the strict application of the inclusion criteria and the quality control of the processed samples. In addition, the multiple Bonferroni correction is an excessively conservative test. Given that the significance level reached in the analyses at the gene level was not far from the threshold, this result could be substantial, especially if we consider that this type of analysis introduces the cumulative effect of rare or low frequency variants. Other potential limitations are the heterogeneity of the study group in terms of age and the categorical distribution of the participants in cases and controls, given that we cannot ensure that none of the controls develop periodontitis in the future. Assuming these limitations, certain results deserve commenting because they might be relevant if confirmed in future studies.

Apparently, the most interesting marker at the SNP level was rs11060842, which is part of gene *PIWIL1*, which in turn is the gene that presents a greater association in the analysis at the gene level. Several studies have shown that *PIWIL1* is overexpressed in various types of cancer, such as lung and endometrial cancer [23,24]. In addition, this gene belongs to the PIWI family, a group of proteins that interact with a class of small RNA specifically expressed in the testicles during spermatogenesis [25], and this gene has been linked with male infertility [26,27]. It has recently been suggested that fertility might be compromised in individuals with DS [28].

The second gene with the greatest level of association was *MIR9-2*. Although *MIR9-2* has not been related to periodontal disease or other oral diseases in the literature, a close association has been reported between this gene and Alzheimer’s disease, whose prevalence is especially high among individuals with DS [29]. *MIR9-2* is directed at two of the most important proteins in the etiopathogenesis of Alzheimer’s disease: the amyloid-beta precursor protein (APP), which transports the amyloid-β peptide that precipitates in amyloid plaques, and β-Site APP cleaving enzyme 1 (BACE1), which cleaves the APP to originate amyloid beta. In addition, *MIR9-2* is expressed differently in brain regions that are significantly associated with the disease’s progression [30].

We should also mention the translocated promoter region (*TPR*) gene, given that the diseases associated with this gene include pulp degeneration [31] and that it has been suggested that chronic periodontitis can affect the dental pulp structure [32].

The pathway that raises the most interest is the PI3K-Akt signaling pathway, which participates in various cell functions, including cell survival and in glucose metabolism, playing an important role in certain infectious diseases [33]. *Porphyromonas gingivalis* produces gingipain, a protein that acts as a toxic protease that destroys the gingival cells, causing chronic periodontitis. Gingipain has been shown to attenuate PI3K activity, favoring the destruction of periodontal tissues mediated by *P. gingivalis* in periodontal diseases [34,35]. It has also been recently confirmed that lipopolysaccharides of the *P. gingivalis* protein have a significant impact on the autophagy of gingival fibroblasts by suppressing the PI3K-Akt-mTOR signaling pathway [36], which participates in the regulation of polymorphonuclear leukocyte activity, given that an increase in PI3K signaling seriously affects chemotaxis and the polarization of neutrophils [37]. Very recent studies conducted with Spanish populations have also confirmed a relationship between rapidly progressive periodontitis and the PI3K-Akt signaling pathway [20].

PI3K pathway dysregulation is one of the most common pathological events in cancer [38] and has been particularly related to leukemia and its therapeutic targets [39,40]. Children with DS have a dramatically higher risk of developing leukemia than the general population, such that this chromosomal disorder is considered the most common risk factor for developing acute lymphoblastic leukemia and acute myeloid leukemia [41].

As has been mentioned, the PI3K-Akt pathway participates in glucose metabolism, activating during hyperglycemia [42]. There is also a greater risk of type 1 diabetes in individuals with DS than in the general population, and this metabolic disorder is often diagnosed at an earlier age in this group [43].

The activity of the PI3K-Akt-mTOR pathway suppresses the autophagy process, while mTOR suppression promotes the process and protects neuronal tissue, which indicates the presence of a coordinated neuroprotective mechanism that is altered in certain neurodegenerative diseases such as Alzheimer’s and Parkinson’s disease [44]. Starting at 40 years of age, most adults with DS develop a neuropathology compatible with Alzheimer’s disease; at 55–60 years of age, at least 70% develop dementia [45]. Periodontitis is more common in the elderly and can be especially prevalent and severe when the ability to maintain adequate oral hygiene is reduced, as is the case for individuals with Alzheimer’s disease [46]. The proliferation of periodontal bacteria causes an increase in proinflammatory serum cytokine levels, and this systemic inflammation state has been associated with a higher rate of cognitive impairment in Alzheimer’s disease [47].

Trisomy 21 causes generalized genetic expression disorders throughout the genome, including continuous activation of the interferon (IFN) transcription response in fibroblasts, lymphoblasts, monocytes, and T cells [48]. Based on the overexpression of IFN receptors encoded in chromosome 21, it has been suggested that DS could be understood as an interferonopathy that causes chronic immune dysregulation [49]. Both type I and type II IFNs regulate the activation of the PI3K-signaling pathway [50]. The analysis of gingival tissue samples from patients with DS and periodontitis showed attenuated expression of signal transducer and activator of transcription 1 (STAT1) and IFN regulatory factor 1 (IRF1) genes, confirming an altered activation of IFN signaling [51].

## 4. Materials and Methods

### 4.1. Study Groups

The study group consisted of 139 individuals with DS, 75 of whom were male, ranging in age from 12 to 53 years (Table S1). This convenience sample was selected among all individuals with DS who regularly attended educational or occupational therapy centers belonging to the Galician Federation of Institutions for Down Syndrome (Spain). All participants satisfied the following inclusion criteria: genetically confirmed diagnosis of DS, age 12 years or older, sufficient degree of collaboration [52] to perform an oral examination and oral sampling and availability of an informed consent signed by the participants or their legal guardians. The exclusion criteria were: age younger than 12 years, subjects unable to cooperate during the periodontal exam [52], coexistence of other systemic diseases that could affect periodontal health (e.g., diabetes), presence of harmful habits (e.g., smoking) and unsigned consent forms. The study was approved by the Research Ethics Committee of Santiago-Lugo (reference 2018/510) and was conducted between November 2018 and December 2019.

To classify the participants by their periodontal biotype, we applied the criteria of the 2017 World Workshop on the Classification of Periodontal and Peri-Implant Diseases and Conditions [53,54], which define periodontitis as a loss of clinical insertion in the vestibular area of ≥3 mm, with sulci >3 mm in 2 or more teeth or a loss of clinical interdental insertion in 2 or more non-adjacent teeth. To apply a case–control design to this study, we selected only those participants with an extreme periodontal phenotype, either with a clear diagnosis of periodontitis (*n* = 52, represented by the cases) or with a healthy periodontal condition (*n* = 36, represented by the controls) (Table S2). The rest of the participants were ultimately excluded (*n* = 51) because they had some degree of gingival inflammation that made it impossible to include them conclusively in any of the 2 defined groups [54].

### 4.2. Collection and Processing of Saliva Samples

Unstimulated saliva samples were taken from all participants using a DNA collection kit (DNA Genotek Inc., Kanata, ON, Canada). The samples were kept stable at room temperature until they were transferred to the Galician Public Foundation of Genomic Medicine (University Clinic Hospital of Santiago de Compostela, Santiago de Compostela, Spain), where the DNA was extracted applying standard protocols (DNA Genotek Inc., 2018). The samples were genotyped using the Axiom Spain Biobank Array (Thermo Fisher Scientific, Waltham, MA, USA), applying the manufacturer’s protocol. This array is a panel that contains 757,836 markers and includes rare, selected variants in the Spanish population (50,536 markers).

To perform the quality control of the genetic markers for these individuals with DS, the analysis excluded chromosome 21 to avoid difficult-to-interpret results. Chromosome 14 was also eliminated, because a large number of cases of DS are due to translocation of genetic material between chromosomes 14 and 21 [55]. A standard quality control of the genotyping data was performed, excluding from the analysis those markers with a genotyping rate less than 98%, applying Plink statistics tool v1.90p [56], and those that deviated from the Hardy–Weinberg equilibrium (*p* < 0.001). Markers were not excluded according to the minor allele frequency (MAF) because they were used in certain analyses [57]. Applying these quality control criteria, we ultimately selected a total of 86 samples (50 cases and 36 controls) and 695,612 markers.

### 4.3. Association Analysis

To test the individual genetic association, we used the case/control status as the dependent variable in a multiple logistic regression analysis performed separately for each SNP (genetic marker showing a MAF > 0.01). In the regression model, we included sex and age as covariates and the SNPx, coded under the dominant genetic model (in which the heterozygotes and homozygotes for the minor allele are grouped and compared with the genotype homozygous for the frequent allele). Only this genetic model was tested to avoid genotypic classes with a low number of observations in cases or controls. The association analysis at this level (SNP by SNP) was performed with Plink v1.9 software [56].

To test the gene-level association, we evaluated the combined effect of common and rare variants by employing the Sequence Kernel Association Test (SKAT) [57], with 3 different approaches: awarding the same weight for all variables (SKAT w1), awarding the weight of the variables by default (SKAT) and applying a simpler collapsing method, the burden test (BURDEN). All options for the SKAT analysis were adjusted for the covariates sex and age. The multitest correction was performed with a Bonferroni threshold that ensured a significance level α of 0.05.

To perform the pathway analysis, we employed the Database for Annotation, Visualization and Integrated Discovery tool v6.8 (DAVID) with which all focus genes with nominal significance (*p* < 0.05) in the analysis at the gene level were tested.

## 5. Conclusions

Given the limitations of this study, we found no SNPs significantly associated with periodontitis in DS at the genome-wide association study level. However, its results suggest that various metabolic pathways are involved in the pathogenesis of periodontitis in DS, including the PI3K-Akt pathway, which regulates cell proliferation and plays a principal role in the host’s inflammatory response. This preliminary report provides a basis for future studies on the genetic susceptibility of individuals with DS for developing periodontitis and details the pathways that are presumably involved.

## Figures and Tables

**Table 1:** Results of individual (SNP by SNP) logistic regression analysis for the top associated SNPs adjusting for covariates.

Marker	Gene	CHR	Minor Allele	N Total	*p*	OR (95% CI)	Genotypes (Dd + dd/DD)
rs4315121	C12orf74	12	T	87	9.85 × 10^−5^	8.84 (3.03–25.77)	PD: 42/9 HP: 15/21
rs4814890	LOC101930064	20	T	87	9.61 × 10^−5^	0.13 (0.05–0.35)	PD: 18/33 HP: 29/7
rs1549874	KBTBD12	3	G	87	8.27 × 10^−5^	0.08 (0.02–0.29)	PD: 4/47 HP: 18/18
rs11060842	PIWIL1	12	C	86	7.82 × 10^−5^	9.05 (2.99–27.33)	PD: 44/7 HP: 16/19
rs62030877	C16orf82(upstr)	16	C	87	8.92 × 10^−5^	0.14 (0.05–0.38)	PD: 10/41 HP: 23/13

CHR, chromosome; OR, odds ratio; CI, confidence interval; DD, frequent homozygotes; (Dd + dd), carriers of the rare allele; PD, periodontal disease (cases); HP, healthy periodontium (controls).

**Table 2:** Genes with major significance after the sequence kernel association test results.

Gene	N Markers (Test)	*p*	SKAT
PIWIL1	47 (44)	1.90 × 10^−5^	SKAT w1
MIR9-2	22 (22)	3.76 × 10^−5^	Burden
LOC101929147	26 (25)	3.93 × 10^−5^	SKAT w1
LHCGR	42 (35)	1.04 × 10^−4^	SKAT
LOC101928304	38 (35)	1.33 × 10^−4^	SKAT
TPR	32 (15)	1.51 × 10^−4^	SKAT w1
BCR	43 (30)	1.55 × 10^−4^	Burden
DERL2	8 (3)	1.76 × 10^−4^	SKAT
CLRN1-AS1	37 (32)	1.97 × 10^−4^	Burden
LOC285501	32 (32)	1.97 × 10^−4^	SKAT
ACVRL1	14 (4)	2.07 × 10^−4^	Burden
PLCXD3	52 (49)	2.50 × 10^−4^	SKAT w1
MIR15A	7 (7)	2.61 × 10^−4^	Burden
AKR1D1	33 (24)	3.03 × 10^−4^	SKAT w1
CDHR4	16 (6)	3.07 × 10^−4^	Burden
LSM8	28 (27)	3.21 × 10^−4^	SKAT w1
CCDC60	96 (86)	3.30 × 10^−4^	SKAT w1
CDCA2	76 (61)	3.32 × 10^−4^	SKAT w1
GNA12	50 (49)	3.34 × 10^−4^	Buden
LOC646762	11 (10)	3.57 × 10^−4^	SKAT w1
COA4	3 (3)	3.70 × 10^−4^	SKAT
MCHR1	17 (15)	4.48 × 10^−4^	SKAT
CACNG8	25 (23)	4.91 × 10^−4^	SKAT
BBS12	44 (23)	4.92 × 10^−4^	SKAT

SKAT, SKAT with “beta” weights; SKAT w1, SKAT with the same weights for common and rare variants; BURDEN, Burden test.

**Table 3:** Pathways with nominal significance after the analysis with DAVID software.

Pathway	Genes (N)	*p*
Long-term depression pathway	15	5.1 × 10^−3^
FoxO signaling pathway	25	1.2 × 10^−2^
PI3K-Akt signaling pathway	53	1.3 × 10^−2^
Glutamatergic synapse	22	1.3 × 10^−2^
Rap1 signaling pathway	35	1.5 × 10^−2^
VEGF signaling pathway	14	1.5 × 10^−2^
Platelet activation	24	1.6 × 10^−2^
Malaria	12	1.7 × 10^−2^
Eph kinases and ephrins support platelet aggregation	5	1.7 × 10^−2^
Fat digestion and absorption	10	2.4 × 10^−2^
Ras signaling pathway	36	2.5 × 10^−2^
T-cell receptor signaling pathway	19	2.6 × 10^−2^
Hepatitis B	25	2.9 × 10^−2^
Wnt signaling pathway	24	3.0 × 10^−2^
Circadian entrainment	18	3.1 × 10^−2^
Primary bile acid biosynthesis	6	3.3 × 10^−2^
Fc epsilon RI signaling pathway	14	3.4 × 10^−2^
Signaling pathways regulating stem cell pluripotency	24	3.4 × 10^−2^
Rho-selective Guanine Exchange Factor AKAP13 Mediates Stress Fiber Formation	5	3.6 × 10^−2^
Fatty acid degradation	10	3.8 × 10^−2^
Wnt signaling pathway	25	4.1 × 10^−2^
TGF-beta signaling pathway	16	4.2 × 10^−2^

## Data Availability

Data available on request due to ethical restrictions.

## Supplementary Materials

The following are available online at https://www.mdpi.com/article/10.3390/ijms22126274/s1.
