# Supplementary material for: Genetic Susceptibility to Periodontal Disease in Down Syndrome: A Case-Control Study
Source: Int J Mol Sci. 2021 Jun 10;22(12):6274. doi: 10.3390/ijms22126274 (PMC8230717; doi:10.3390/ijms22126274)
Supplement: Supplementary file 1 [file ijms-22-06274-s001.zip › ijms-1235679-supplementary.pdf]

Supplementary Material

Table 1. Patient sample characteristics (age, sex and systemic conditions).

		Full Sample	Extreme Phenotype (Included in the Study)		Gingivitis (Excluded from the Study)
			**Cases with Periodontitis**	**Controls with Healthy Periodontium**	
**Age (years)**	**Mean**	24.9	27.2	26.8	21.4
**Sex**	**Female, *n* (%)**	64 (46)	18 (34.6)	17 (47.2)	29 (56.9)
	**Male, *n* (%)**	75 (54)	34 (65.4)	19 (52.8)	22 (43.1)
**Systemic diseases**	**All conditions, *n* (%)**	24 (17.3)	10 (19.2)	11 (30.5)	3 (5.9)
	**Heart disease, *n* (%)**	9 (6.5)	5 (9.6)	1 (2.8)	3 (5.9)
	**Thyroid disease, *n* (%)**	19 (13.7)	2 (3.8)	10 (27.8)	7 (13.7)
	**Thyroid medication, *n* (%)**	19 (13.7)	2 (3.8)	10 (27.8)	7 (13.7)
	**Other conditions, *n* (%)**	8 (5.8)	3 (5.8)	3 (8.3)	2 (3.9)

Table 2. Periodontal clinical outcome variables for the whole sample (mean±standard deviation).

	Full Sample	Extreme Phenotype (Included in the Study)		Gingivitis (Excluded from the Study)
		**Cases with Periodontitis**	**Controls with Healthy Periodontium**	
**PD**	2.39 ± 0.47	2.72 ± 0.47	2.07 ± 0.11	2.13 ± 0.19
**REC**	0.08 ± 0.25	0.14 ± 0.35	0.02 ± 0.06	0.02 ± 0.04
**PII**	56.5 ± 31.5	71.2 ± 25.8	41.8 ± 30.3	58.3 ± 26.7
**BOP**	19.9 ± 17.8	32.2 ± 16.7	7.7 ± 7.7	25.3 ± 9.2

Abbreviations: PD, probing depth; REC, gingival recession; PII, plaque index; BOP, bleeding on probing
